# Contribution of Active Surface of NiFe-Layered Double Hydroxide on the Removal of Methyl Orange

**DOI:** 10.3390/ma18040911

**Published:** 2025-02-19

**Authors:** Yanping Zhao, Fengzhu Lv, Yanwen Ou, Guocheng Lv, Shifeng Zhao

**Affiliations:** Engineering Research Center of Ministry of Education for Geological Carbon Storage and Low Carbon Utilization of Resources, Beijing Key Laboratory of Materials Utilization of Nonmetallic Minerals and Solid Wastes, National Laboratory of Mineral Materials, School of Materials Science and Technology, China University of Geosciences (Beijing), Beijing 100083, China; ypzhao0423@163.com (Y.Z.); ouyanwen2022@163.com (Y.O.); zsf17865932389@163.com (S.Z.)

**Keywords:** NiFe-layered double hydroxide, active surface, synergistic effect, vacancy defects

## Abstract

Layered double hydroxides (LDHs) have potential applications for pollutant removal. Enhancing their pollutant removal ability by fully utilizing the synergistic effects of physical adsorption and chemical catalysis has received widespread attention. In this study, a high methyl orange (MO) removal capacity was achieved by utilizing the synergistic effects of physical adsorption and chemical catalysis of NiFe-LDH. wNiFe-LDH showed a significant removal amount of MO, up to 506.30 mg/g due to its reserving of the active surface to the largest extent. Experiment and molecular simulation clarified the high removal capacity derived from surface adsorption and the degradation ability of the active surface. The presence of more -OH groups on the surface enhanced the removal of MO, and the vacancies in the surface were beneficial for the formation of •O_2_^−^ and contributed to the degradation of MO. As K_2_S_2_O_8_ was introduced, the removal rate of MO improved to 100% from 60.67%. However, a deeper study showed that the degradation was incomplete, as K_2_S_2_O_8_ inhibited the formation of •O_2_^−^, and the active species in the system changed to holes. The degradation path of MO was also altered. Thus, this study gives new insight into the reactivity of the active surface of NiFe-LDH and affords a new path to preserve the active surface.

## 1. Introduction

In recent years, the threat of organic waste to the environment has become more and more serious [1,2,3,4,5]. The preparation of high-efficiency water treatment materials was an urgent task. Inorganic clay mineral materials such as bentonite, sepiolite, and attapulgite have high specific surface areas and abundant pore structures [6] and can adsorb organic molecules effectively [7]. Layered mineral materials have gained more attention due to their wide availability [8], low cost, and ease of application on a large scale [9].

Hydrotalcite is a natural mineral with a positive charge and layered structure, which shows great potential in organic wastewater treatment [10]. As a result of structure and composition adjustment based on hydrotalcite, layered double hydroxides (LDHs) not only showed high adsorption capacity but also exhibited catalytic degradation function toward organic pollutants [11] due to their adjustable interlayer space, layer charge, and composition [12,13]. Therefore, using the synergistic effect of adsorption and degradation to treat organic wastewater has become a research hotspot [14]. For example, studies have shown that CoAl-layered double hydroxide (LDH) nanosheets can effectively remove organic pollutants from water (>90%) [15]. In another study, the nano-confined catalytic membrane developed from CoFeCu-LDH demonstrated excellent water purification performance, realizing efficient removal of a variety of micropollutants [16]. Compared with the commonly studied catalytic materials (Table S1 in Supplementary Materials) [17,18,19,20,21,22,23,24], LDH shows great advantages in high concentration and large-scale organic pollutant removal.

Defect engineering, whether intrinsic defects or extrinsic defects, is regarded as an effective strategy to modulate the electronic properties of materials and create active centers [25]. For LDHs, creating vacancy defects to regulate the electronic structure is an important method. Preparing monolayer LDHs could expose a large number of active sites, but the tendency of monolayer LDHs to agglomerate greatly affected the stability of their catalysts [26]. Wang [27] published a study utilizing plasma technology to strip LDHs in situ under a nitrogen atmosphere. The technology could not only create a large number of defects and increase the active sites but also change the electronic structure around the active sites, accelerating electron transport. However, the strategy could not be used in conventional preparation processes, where active sites were buried in the drying process. Therefore, creating and utilizing the active surface remains difficult. As shown in Table S1, LDH has significant advantages in the removal of methyl orange because LDH is not prone to crystallization, which is conducive to adsorption and degradation. As shown in Table S1, LDH had significant advantages in the removal of methyl orange because LDH was not prone to crystallization, which facilitates adsorption and degradation.

NiFe-LDH, relying on its unique layered structure, high catalytic activity, and abundant active sites [28], has been studied in the field of catalytic degradation [29]. NiFe-LDH could be modified by lattice doping, vacancy defects, and surface modification to improve its catalytic performance [30], thus showing good performance in the degradation of methyl orange and Congo red [31].

In addition, the adsorption and catalytic performance of NiFe-LDH could be optimized by adjusting its metal cation ratio and interlayer anions [32]. Although NiFe-LDH showed potential for the degradation of organic matter, some issues still need to be addressed. There is still room for improvement in the degradation effect. At the same time, the degradation mechanism of NiFe-LDH for organic pollutants still needs to be further clarified, especially regarding the research on the reaction mechanism of its active surfaces.

Hence, in the present work, the contribution of the active surface of NiFe-LDH to the removal of MO was methodically investigated. As the active surface was reserved to the largest extent, the MO removal amount could reach up to 506.30 mg/g due to adsorption and degradation effects. Systematic experiments and molecular simulation calculations for system energy and electron density were applied to clarify the mechanism. The function of -OH and vacancies on the surface of NiFe-LDH was the key focus of the study. In addition, many strategies were applied to improve the amount of MO removal. Different reaction mechanisms were determined as K_2_S_2_O_8_ was introduced in different addition orders. When K_2_S_2_O_8_ first touched the NiFe-LDH, the high activity of K_2_S_2_O_8_ hindered the production of •O_2_^−^ and altered the degradation path of MO. Thus, controlling the reaction state of the active surface was important for the removal of MO. Therefore, the present work provided insight into active surface reservation and utilization.

## 2. Experimental Part

### 2.1. Materials

NiCl_2_·6H_2_O, FeCl_3_·6H_2_O, NaOH, hydrochloric acid, and K_2_S_2_O_8_ were all purchased from Xilong Chemical Co., Ltd. (Shantou, China). MeOH was supplied by Sigma Chemical Reagent Co., Ltd. (Dorset, UK). Triethanolamine (TEA), tert-butanol (TBA), isopropanol (IPA), benzoquinone (BQ), and methyl orange (MO) were purchased from Beijing Chemical Reagent Factory (Beijing, China). These reagents were all analytical grade and used without any further purification.

### 2.2. Synthesis of NiFe-LDH

#### 2.2.1. NiFe-LDH Prepared by Co-Precipitation Method

A series of NiFe-LDH with different molar ratios of Ni to Fe (4:1, 3:1, and 2:1) was prepared by co-precipitation. To prepare NiFe-LDH with Ni:Fe = 4:1, NiCl_2_·6H_2_O (0.48 g) and FeCl_3_·6H_2_O (0.14 g) were mixed in 100 mL of deionized water. Subsequently, NaOH solution (0.24 g/50mL) was added dropwise into the above solution under continuous stirring to adjust the pH to 9–10. After 30 min of reaction, the mixture was aged for 24 h and then centrifuged. Finally, half of the obtained precipitate was dried at 60 °C for 48 h and assigned as dNiFe-LDH; the other half was dispersed in 20 mL of deionized water to maintain the active surface of NiFeLDH and assigned as wNiFe-LDH.

#### 2.2.2. Preparation of Single-Layer NiFe-LDH

NiCl_2_·6H_2_O (0.475g) and FeCl_3_·6H_2_O (0.135 g) were mixed in 100 mL of deionized water. Then, 20 mL of formamide was added. Subsequently, NaOH solution (0.24 g/50 mL) was added dropwise into the above solution under continuous stirring to adjust the pH to 9–10. Thirty minutes later, the mixture was aged for 24 h and then centrifuged. Finally, the obtained precipitates were washed three times using deionized water and then dispersed in 20 mL of deionized water. The sample was assigned as wNiFe-sLDH.

### 2.3. Removal of MO by NiFe-LDH

#### 2.3.1. Removal of MO by NiFe-LDH Prepared Based on Co-Precipitation Method

To study the removal ability of NiFe-LDH, MO solution with concentrations of 50, 150, 250, 350, 450, and 550 mg/L was prepared. Then, 2 mL of wNiFe-LDH suspension was added to 25 mL of MO solution. After a reaction for 30 min, the mixture was centrifuged, and the absorbance of the supernatant at 465 nm was tested. The precipitates were dried to form a series of samples named wNiFe-MO-x (x = 50, 150, 250, 350, 450, 550).

#### 2.3.2. Removal of MO in the Preparation Process of NiFe-LDH (One-Step Method)

A total of 0.48 g of NiCl_2_·6H_2_O and 0.14 g of FeCl_3_·6H_2_O were mixed in 50 mL of deionized water. Then, 50 mL of methyl orange (MO) solution (250 mg/L) was introduced. Subsequently, NaOH solution (0.24 g/50 mL) was added dropwise into the above solution under continuous stirring to adjust the pH to 9–10. The removal process was maintained for 30 min. To study the removal rate, the final precipitate was collected and assigned as MO-wNiFe-LDH. If aging for 24 h was applied after the addition of the NaOH solution, the given precipitate was assigned as MO-wNiFe-LDH-a.

### 2.4. Strategy Study on the Removal Rate Improvement

To improve the removal rate of MO with wNiFe-LDH, visible light/ultraviolet light/microwave/ultrasound were applied to the addition process of NaOH. Also, K_2_S_2_O_8_ was added to the reaction system to improve the removal rate.

The order of the introduction of reaction components was studied in relation to the influence of K_2_S_2_O_8_. To ensure that the active surface of NiFe-LDH first came into contact with different components at the beginning of the reaction, three suspensions were prepared: K_2_S_2_O_8_ + MO, K_2_S_2_O_8_ + wNiFe-LDH, and wNiFe-LDH + MO, after which the third component was introduced. The final products in the three cases were assigned as S-MO-wNiFe, S-wNiFe-MO, and wNiFe-MO-S, respectively.

The concentration of MO in this series of studies was 250 mg/L, and the dosage of K_2_S_2_O_8_ was 10 mg.

### 2.5. Chemical Quenching Experiments

Chemical quenching experiments were conducted to identify the contributions of reactive species to MO degradation. MeOH, IPA, BQ, TEA, and PB were selected as the scavengers of •SO_4_^−^, •OH, •O_2_^−^, h^+^, and e^−^, respectively [33].

### 2.6. Characterization

The X-ray diffraction patterns were recorded using a Rigaku D/Max-rA rotating anode X-ray diffractometer (Rigaku, Tokyo Metropolis, Japan) equipped with a Cu Kα radiation (λ = 0.1542 nm). Patterns were measured for all samples from 5 to 75° with a step size of 0.02°. The morphology of the materials was examined using scanning electron microscopy (Cobra FIB FE-SEM, Auriga, Oberkochen, Germany). The pollutant removal properties were recorded using a UV spectrophotometer, model UV-600 (Thermo Fisher Scientific, Waltham, MA, USA), and each data point represented the average of four tests. The standard curve of MO was drawn from Figure S1a and shown in Figure S1b. The removal amount was calculated based on Formula (1).

(1) $$Removal\;amount=\frac{{(C}_{0}-C_{t})V}{m_{wNiFe-LDH}}$$

Among these, C_0_ and C_t_ were the concentrations of MO solution at the beginning and at time t (mg/L). V was the volume of MO solution and $m_{wNiFe-LDH}$ was the weight of NiFe-LDH.

The thermal stability of the samples was analyzed by a Thermogravimetric Analyzer (TGA) (SDT Q600, TA Instruments, New Castle, DE, USA). The degradation pathway was recorded using an in situ infrared spectrometer (in situ FTIR) (Nicolet iS50, Thermo Scientific, Waltham, MA, USA). The software Materials Studio (Materials Studio 2023), with CASTEP and Forcite, was used for computer molecular simulation.

## 3. Results and Discussion

### 3.1. Preparation and Characterization of NiFe-LDHs

**Figure 1:**
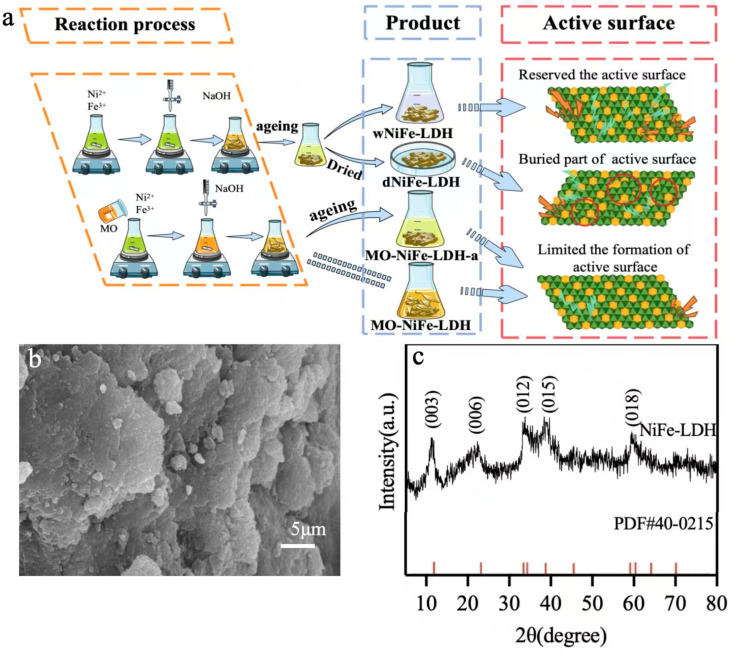
(**a**) Preparation of four types of NiFe-LDH; (**b**) SEM image of wNiFe-LDH; (**c**) XRD patterns of wNiFe-LDH.

Four kinds of NiFe-LDHs with different contents of active surface were prepared based on the routine shown in Figure 1a. The active surface of wNiFe-LDH was reserved at the largest content by omitting operations that promoted unfavorable single-layer LDH formation and was used to treat MO in situ. For comparison, dNiFe-LDH and NiFe-LDH-a were prepared by undergoing only aging or drying, which promoted the stacking of LDH layers. Also, MO-NiFe-LDH was prepared under the covering effect of MO on the surface. As the natural state of wNiFe-LDH could not be characterized using general methods, dNiFe-LDH was characterized in detail and is shown in Figure 1b,c, which represents samples conserving the structure of wNiFe-LDH. The SEM image of the wNiFe-LDH indicated that it was a tightly stacked layered structure. The diffraction peaks at 2θ = 11.6, 22.7, 33.8, 38.9, 46.5, and 60.5° in the XRD pattern of wNiFe-LDH could be assigned to the (003), (006), (012), (015), (018), and (110) planes of typical LDH frameworks (PDF#40-0215) [17,34]. However, the lower diffraction peak intensity implied poor crystallinity of wNiFe-LDH. These results indicated that NiFe-LDH was successfully prepared.

### 3.2. Removal Efficiency of MO by NiFe-LDH

#### 3.2.1. Removal Efficiency of MO by NiFe-LDH with Different Content of Active Surface

**Figure 2:**
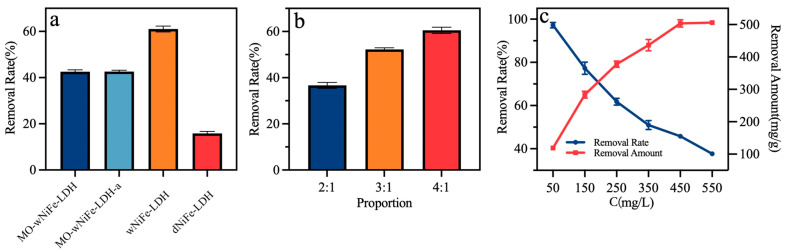
(**a**) Removal rate of MO (50 mg/L) by NiFe-LDH with different active surface areas; (**b**) removal rate of MO (50 mg/L) by wNiFe-LDH with differentNi/Fe molar ratios; and (**c**) removal rate under different pH conditions and initial MO concentrations.

In order to investigate the contribution of the active surface of NiFe-LDH to MO removal, four strategies were applied to maintain different contents of active surfaces. As shown in Figure 1a and Figure 2a, when NiFe-LDH was dried and some active surface was buried due to drying, the removal rate of NiFe-LDH for 250 mg/L of MO was only 15.52%. However, when the newly forming NiFe-LDH was maintained in a wet state, the removal rate of MO reached 60.67%, representing an improvement of about 3.9-fold. When MO was introduced during the formation process of NiFe-LDH, although the wet state was maintained, the removal rate was nearly 42%, whether aged or not, which may be due to the limiting effect of MO on the active surface formation. Thus, maintaining the active surface only by maintaining the formation precipitates in the wet state could greatly improve the removal ability of NiFe-LDH. Further research was therefore carried out on adjusting the element ratio of wNiFe-LDH.

#### 3.2.2. Effect of Ni/Fe Ratio and Initial Concentration on Removal Rate of MO

As shown in Figure 2b, the molar ratio of Ni to Fe had a significant effect on the removal rate of MO (250 mg/L) [35]. When the molar ratios of Ni to Fe were 2:1, 3:1, and 4:1, the removal rates of MO were 36.73%, 51.55%, and 60.67%, respectively. wNiFe-LDH (Ni:Fe = 4:1) had the highest removal rate of MO. Therefore, wNiFe-LDH was selected for subsequent testing, and its structural formula was Ni_0.8_Fe_0.2_(OH)_2_.

Initial concentration was an important factor that affected the removal rate. When the initial concentration was 50 mg/mL, an almost 100% removal rate could be reached. With the increase in the initial concentration of MO, the removal rate of MO decreased, but the removal amount increased, as shown in Figure 2c, and the data are summarized in Table S2. When the initial concentration was 450 mg/L, the maximum removal amount reached 506.30 mg/g. In the literature, the removal amounts of NiFe layered double hydroxide nanoflakes decorated by montmorillonite (MMT@NiFe LDH) and S/NiFe-LDH (1:1), which had similar structures to the sample in the present work, were 108.80 mg/g and 246.91 mg/g [17,36]. Thus, the removal of MO by NiFe-LDH was significantly improved in the present work due to the maintenance of the active surface.

### 3.3. Removal Mechanism of MO with wNiFe-LDH

#### 3.3.1. Thermal Analysis and Reaction Species Identification

To determine how MO was removed, TGA analysis was performed. Thermogravimetric analysis (TGA) was used to measure the mass changes of materials during heating. Since MO completely decomposed at around 300 °C, while NiFe-LDH remained relatively stable, this allowed the adsorbed amount of MO in the sample after the reaction to be calculated by comparing the weight loss rate of the samples before and after the reaction. This allowed for the determination of the adsorption and degradation rates of methyl orange by NiFe-LDH.

**Figure 3:**
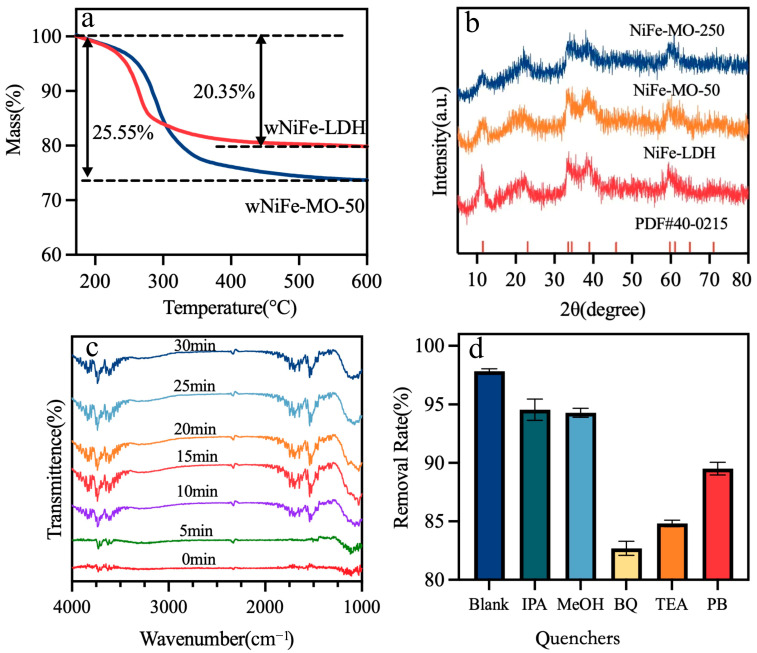
(**a**) TGA curves of wNiFe-LDH and wNiFe-MO-50; (**b**) XRD patterns of wNiFe-LDH, wNiFe-MO-50, and w NiFe-MO-250; (**c**) in situ FTIR spectra of the MO solution; and (**d**) removal rate of MO under different quenching conditions.

Figure 3a shows the TGA of wNiFe-LDH and wNiFe-MO-50. From the thermal decomposition curve of wNiFe-LDH, it can be inferred that wNiFe-LDH began to decompose significantly at about 250 °C, and the weight loss from 150 °C to 800 °C was about 20.35%. The weight loss of wNiFe-MO-50 at the same temperature range was 25.55%. Also, the decomposition temperature of wNiFe-MO-50 increased as the decomposition temperature of MO was at about 300 °C. These phenomena indicated that there were MO residues in wNiFe-MO-50. Since MO completely decomposed at 300 °C, the remaining residue (74.45%) corresponded solely to the calcined products of NiFe-LDH, which were the oxides of Ni and Fe. Thus, the weight loss of 25.55% resulted from the decomposition and weight change of NiFe-LDH into its oxides. By deducting the contribution of weight change of NiFe-LDH to its oxides, the adsorbed amount of MO on NiFe-LDH was calculated. Further subtracting the adsorbed amount from the total MO removal yielded the degraded fraction. This method effectively distinguished adsorption from degradation contributions. Accordingly, the adsorbed and degraded MO were about 44.86% and 55.14%, respectively. These observations evidently confirmed that part of MO degraded and the other part was adsorbed by wNiFe-LDH.

XRD tests were conducted to determine the adsorption position of MO. As shown in Figure 3b, lower diffraction peak intensity implied poor crystallinity of wNiFe-LDH, which was beneficial for adsorption. For clearer observation, a higher removal amount sample, wNiFe-MO-250, was also tested. The diffraction peaks of wNiFe-MO-50 and wNiFe-MO-250 showed no deviation compared with wNiFe-LDH, indicating that no MO was intercalated into the interlayer of wNiFe-LDH layers. Thus, MO was just adsorbed on the surface. Therefore, the removed MO involved non-intercalation adsorption and degradation.

In situ FTIR (Figure 3c) of the reaction mixture revealed the degradation pathways of MO. In the initial mixture, the stretching vibration peak of the aromatic ring could be observed at 1525 cm^−1^. As the reaction proceeded for 15 min, a series of peaks in the range of 1260–2000 cm^−1^ appeared, clearly indicating the degradation of MO. Molecular simulation was applied to study the possible breaking position of MO. The calculated bond lengths of MO are shown in Table S3. The C-S bond was the longest one which was the easiest position to break. However, the bond cleavage energy barrier of the -N=N- bond in methyl orange was 1.2 eV, which was lower than that of the -C-S- bond cleavage (1.8 eV), indicating the preferential cleavage of the azo bond. So, the possible breaking position at the initial period was -N=N- bond. Also, the infrared adsorption peak of CO_2_ was observed at 2335 cm^−1^ [37], indicating that the intermediates were further broken into small inorganic molecules. The benzene ring-opening pathway to generate CO_2_ exhibited an overall ΔG of −2.5 eV. In summary, the degradation of MO began at 10 min, and the initial breaking location could be -N=N- and the benzene ring connected with sulfonate.

Chemical quenching experiments were conducted to identify the contributions of reactive species (•SO_4_^−^, •OH, •O_2_^−^, h^+^, and e^−^) to MO degradation (Figure 3d). CH_3_OH, IPA, BQ, TEA, and PB were selected as the scavengers of •SO_4_^−^, •OH, •O_2_^−^, h^+,^ and e^−^, respectively. CH_3_OH, IPA, and PB showed little influence on MO removal, indicating that •SO_4_^−^, •OH, and e^−^ were not largely produced in the wNiFe-LDH/MO system. The degradation of MO decreased significantly with the addition of BQ in the wNiFe-LDH/MO system, indicating that •O_2_^−^ might play an important role in MO degradation. By adding TEA, ~10% inhibition of MO removal efficiency was observed, which showed that h^+^ was also involved in MO degradation. In summary, •O_2_^−^ and h^+^ were the active species in the present system.

#### 3.3.2. Insights into the Contribution of -OH Groups and Vacancy on the Active Surface

Generally, newly formed surfaces could preserve defects to the greatest extent, and vacancies were a major manifestation of crystal defects. Thus, -OH groups and vacancies were the main components of the active surface of NiFe-LDH.

**Figure 4:**
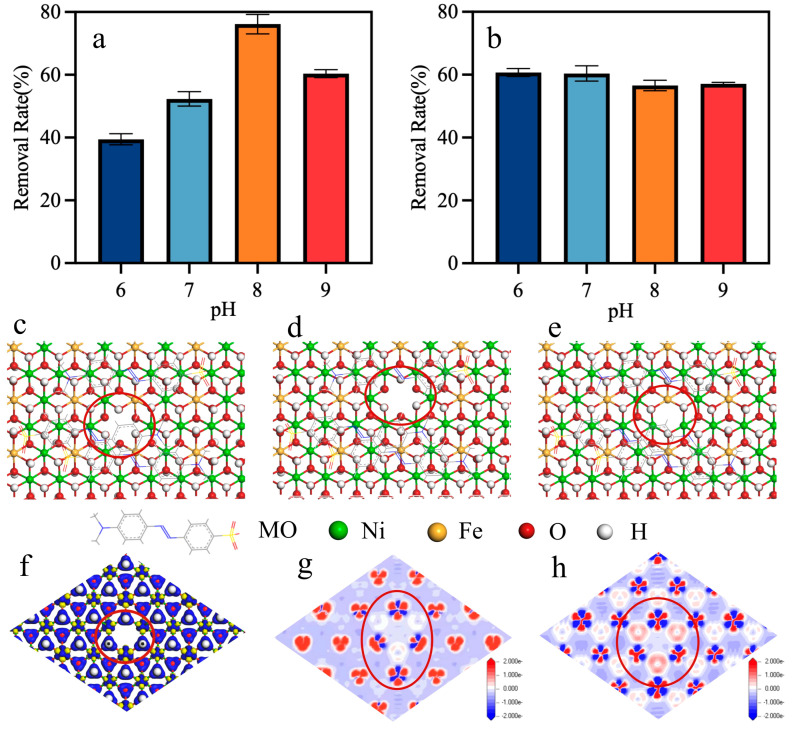
Removal rate of MO at different pH for (**a**) wNiFe-LDH suspension and (**b**) wNiFe-LDH/MO mixture. Diagrams of wNiFe-LDH-MO with (**c**) Ni vacancy, (**d**) Fe vacancy, and (**e**) O vacancy (as shown in the position of the red circle) on the surface of wNiFe-LDH. Charge density of (**f**) NiFe-LDH with Vo, (**g**) Ni-Fe slice, and (**h**) Vo slice.

To determine the effects of hydroxyl groups on the active surface of wNiFe-LDH on MO removal, the reaction properties of -OH on the NiFe-LDH surface were mediated. Figure 4a shows the removal rate of MO after adjusting the pH of wNiFe-LDH suspension first to endow the surface of LDH with different charges and then using it to treat MO. As shown in Figure S2, the characteristic peaks of MO were not affected by pH. Thus, different pH levels represented different contents of -OH groups and charges on the surface. When the pH of the suspension decreased from 8 to 6, the removal rate of MO decreased from 76.42% to 52.83%, and then to 39.19% sequentially, implying that decreasing the amount of -OH on the surface restrained the removal of MO. Further increasing the pH to 9 still resulted in a lower MO removal rate. This pH-dependent behavior highlighted the critical role of surface charge in adsorption efficiency. NiFe-LDH typically exhibited a positively charged surface in aqueous solutions due to the protonation of surface hydroxyl (-OH) groups. Since MO was an anionic dye, strong electrostatic attraction enhanced its adsorption onto the positively charged NiFe-LDH surface. At higher pH (pH = 9), NiFe-LDH became negatively charged, leading to electrostatic repulsion with MO and reduced adsorption. However, when the pH of the wNiFe-LDH/MO mixture was adjusted to 6, 7, 8, or 9, the removal of MO showed little difference (Figure 4b), remaining at ~60%. As the surface of wNiFe-LDH had been covered by MO, the adjustment of pH had little influence on the state of surface -OH. Thus, the MO removal difference at different pH levels derived from the charge and -OH amount difference on the wNiFe-LDH surface. These results provided favorable evidence that the charge or the amount of -OH on the surface of wNiFe-LDH affected the removal.

It was assumed that -OH existed as OH_2_^+^, -OH, or -(OH)_2_ in acidic, neutral, and strongly alkaline environments. To determine the influence of different states of -OH, the system energy of LDH with MO was calculated when part of OH was changed to OH_2_^+^ or -(OH)_2_. Higher energy indicated less stability. Thermodynamic calculations indicated that the structure of NiFe-LDH with OH_2_^+^ on its surface could not exist stably. However, as -OH was replaced by -(OH)_2_, the system energy of NiFe-LDH decreased from 12.28 kcal/mol to 12.19 kcal/mol. So, a more stable structure was formed, which was unfavorable for MO adsorption. Therefore, as more -OH existed in a neutral state, a higher MO removal rate was realized, which might be due to adsorption.

In order to further clarify the removal mechanism of MO by the active surface and track the production origin of active species, three kinds of vacancies were fabricated, and the binding energy of MO with wNiFe-LDH containing different vacancies was calculated by simulation. The vacancies studied were Ni vacancy (Figure 4c), Fe vacancy (Figure 4d), and O vacancy (Vo) (Figure 4e), respectively. The binding energy of MO with wNiFe-LDH notably decreased from 12.28 kcal/mol to 0.61 kcal/mol, 10.62 kcal/mol, and 10.32 kcal/mol, respectively. Higher binding energy indicated poor stability of the system. Thus, the surface of wNiFe-LDH with vacancies was not conducive to the adsorption of MO.

Charge density change was another important result of vacancy formation. Thus, the charge density change was calculated when Vo was present. As shown in Figure 4f, removing one oxygen atom changed the charge density around the vacancy. In order to better display charge density changes, a slice passing through nickel-iron atoms and a slice in a horizontal plane with Vo were shown. It was clear that the charge density around Ni and Fe declined, showing an electron-deficient state (Figure 4g). However, the charge density around Vo increased (Figure 4h). This indicated that Vo had the ability to accumulate and redistribute electrons from metals in the catalyst [38], making a considerable amount of electrons accumulate around the vacancy, which endowed an enhanced ability to lose electrons. Similar results were obtained when Ni vacancy and Fe vacancy were fabricated [39]. Thus, the existence of vacancies on the surface of NiFe-LDH contributed to the formation of •O_2_^−^ as well as the degradation of MO.

#### 3.3.3. Removal Mechanism of MO with wNiFe-LDH

**Figure 5:**
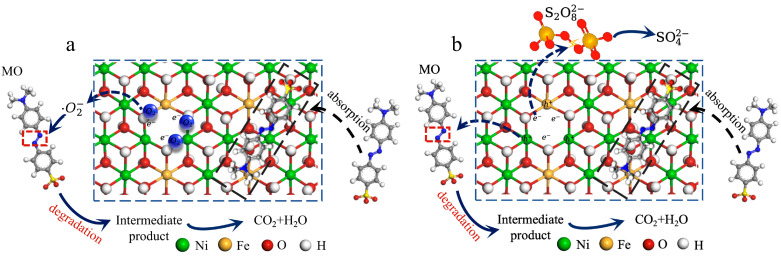
Illustration of the removal mechanism of MO (**a**) with wNiFe-LDH and (**b**) in the S-wNiFe-MO system.

Based on the results and discussion above, the removal mechanism of wNiFe-LDH for MO was proposed in Figure 5a. The removal was divided into two parts. Half of the MO was adsorbed on the surface of wNiFe-LDH by electrostatic force. The other half was degraded by free radicals, especially by •O_2_^−^. •O_2_^−^ was produced by the reaction of O_2_ adsorbed onto the surface, which gained the lost electron from elements around the vacancy [40,41,42,43,44]. Then, active •O_2_^−^ further attacked MO, breaking it down into intermediate products, and finally into CO_2_ and H_2_O.

To verify the mechanism of MO degradation, single-layer NiFe-LDH, which theoretically had the greatest active sites on its surface, was prepared by adding formamide to the system. The removal rate of MO by single-layer NiFe-LDH was significantly increased to 80.54% from 60.67%. This positive result demonstrated the catalytic activity of wNiFe-LDH, strongly verifying the degradation effect of the newly generated surface of wNiFe-LDH.

### 3.4. Further Improvement of MO Removal Rate

#### 3.4.1. Strategy Study on the Removal Rate Improvement

**Figure 6:**
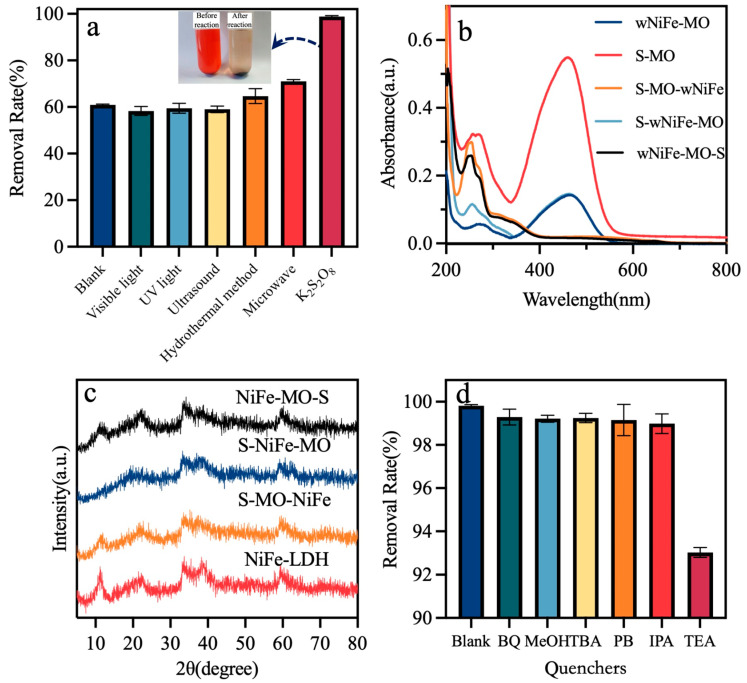
(**a**) Removal rate of MO under different supplementary assistants; (**b**) influence of ingredient addition order on removal rate; (**c**) XRD pattern of wNiFe-LDH, S-MO-wNiFe, S-wNiFe-MO, and wNiFe-MO-S; and (**d**) free radical trapping results of S-wNiFe-MO.

To improve the removal of MO by wNiFe-LDH, visible light/ultraviolet light/ultrasound/the hydrothermal method/microwave treatment and K_2_S_2_O_8_ were chosen to assist the reaction based on the literature [45,46,47]. The results are shown in Figure 6a. A higher MO concentration was selected to better observe the concentration change and the influence of different factors. When the concentration of MO was 250 mg/L, visible light/ultraviolet light/ultrasound did not affect the removal of MO. The hydrothermal method and microwave treatment slightly improved the removal efficiency from 60.67% to 65.24% and 70.48%, respectively. Notably, the addition of K_2_S_2_O_8_ greatly enhanced the removal rate of MO to 100%. As reported in previous studies, peroxymonosulfate was activated to produce highly oxidative sulfate and hydroxyl radicals, which attacked the azo bonds and aromatic rings in methyl orange, thereby facilitating its degradation. In order to clarify the interaction of K_2_S_2_O_8_ with the active surface_,_ three reaction procedures were designed. In the first case, the active surface first came into contact with K_2_S_2_O_8_. In the second case, the active surface came into contact with a complex of K_2_S_2_O_8_ and MO. The third case was that the active surface first came into contact with MO. In the second and third cases, the removal rate of MO increased to 100%, but the removal rate in the first case was only 60.29%, remaining basically unchanged compared to that without K_2_S_2_O_8_ (Table 1). So, it is inferred that the active surface should first come into contact with MO, and then K_2_S_2_O_8_ takes effect.

However, the UV-visible absorption diagram (Figure 6b) of the MO in the three cases changed greatly. As K_2_S_2_O_8_ worked or did not come into contact with the active surface of LDH directly, after a 30 min reaction, the absorption peak at 465 nm disappeared completely. However, the absorption intensity at 279 nm was almost unchanged. The absorption peak at 465nm was generated by the n-π* transition from the azobenzene structure in MO [48]. Thus, the introduction of K_2_S_2_O_8_ only changed the degradation path of MO; more intermediates were produced. Studies have shown that NiFe-LDH can catalyze or activate persulfate to degrade organic matter [36,49,50,51,52]. However, when persulfate came into contact with the LDH surface first, the removal of MO was not influenced. Therefore, further investigation is needed to clarify this phenomenon.

As shown in Figure 6c, when K_2_S_2_O_8_ first comes into contact with the active surface of LDH, the diffraction peak at 11.6 in the XRD pattern of the product (S-wNiFe-MO) disappears completely. In the other two cases, the crystalline structure of the products (wNiFe-MO-S and S-wNiFe-MO) remains similar to that of wNiFe-LDH. Thus, the interaction between K_2_S_2_O_8_ and the active surface of wNiFe-LDH occurs as they first come into contact, which is not the preferred method for removing MO.

To clarify the degradation path of MO when K_2_S_2_O_8_ was active, chemical quenching experiments were conducted to identify the contributions of reactive species (•SO_4_^−^, •OH, •O_2_^−^, h^+^, and e^−^) (Figure 6d). MeOH, BQ, TEA, and PB were selected as the scavengers of •SO_4_^−^, •O_2_^−^, h^+^, and e^−^, respectively, while IPA and TBA were used to capture •OH. The removal rate of MO decreased by ~7% with the addition of TEA, revealing that h^+^ in the system played a significant role. Other radical scavengers had no effect on MO removal, and •SO_4_^−^ was not detected as expected.

#### 3.4.2. Removal Mechanism with the Effect of K_2_S_2_O_8_

Based on the results and discussion above, the removal mechanism was proposed in Figure 5b as K_2_S_2_O_8_ affected the degradation. However, adsorption and degradation both contributed to the removal of MO. Due to the enrichment of electrons at the oxygen vacancy [53], the excess electrons were easily captured by K_2_S_2_O_8_, producing SO_4_^2-^, as K_2_S_2_O_8_ is a strong oxidant. The depletion of electrons exposed the holes, which resulted in the degradation of MO. Since the attacking ability of holes and •O_2_^−^ differs, under attack by holes alone, the molecule fragmented, leaving the substituent groups linked to the benzene ring. Thus, benzene rings remained in the solution as intermediates. In the presence of K_2_S_2_O_8_, the degradation of MO was incomplete.

In summary, the present work provides a method to effectively remove MO by maintaining the active surface of NiFe-LDH. However, the question of how to preserve the active surface and maintain high removal efficiency in large-scale applications and at remote pollutant sites still needs to be addressed.

## 4. Conclusions

In the present work, newly produced NiFe-LDH with an active surface, which was preserved to the greatest extent by maintaining the products in a wet state, was used to remove MO. Under optimum conditions, wNiFe-LDH could achieve a 100% removal rate for 50 mg/mL of MO solution, and its removal capacity could reach up to 506.30 mg/g. The removal included surface adsorption and degradation. Experiment and molecular simulation were used to confirm the contribution of -OH and vacancy on the active surface. On a neutral surface, more -OH contributed to higher MO removal. DET calculation confirmed the electron enrichment around vacancies on the surface of wNiFe-LDH, favorable for the formation of •O_2_^−^ and conducive to the degradation of MO. With the addition of persulfate, when persulfate did not come into contact with the active surface of wNiFe-LDH, it could improve the MO removal rate from 60.64% to 100%. However, the degradation of MO in this case was incomplete, and intermediates containing benzene rings were produced. The introduction of persulfate captured the electrons around vacancies and changed the active species from •O_2_^−^ to holes, which further changed the degradation path of MO.

## Figures and Tables

**Table 1:** Removal rate of S-MO, wNiFe-MO, S-MO-wNiFe, wNiFe-MO-S, and S-wNiFe-MO.

Samples	C After Reaction (mg/L)	Removal Rate (%)
S-MO	202.15	19.14
wNiFe-MO	98.33	60.67
S-wNiFe-MO	99.28	60.29
S-MO-wNiFe	/	100
wNiFe-MO-S	/	100

## Data Availability

The original contributions presented in the study are included in the article and Supplementary Materials. Further inquiries can be directed to the corresponding authors.

## Supplementary Materials

The following supporting information can be downloaded at: https://www.mdpi.com/article/10.3390/ma18040911/s1, Figure S1:(a) Ultraviolet spectra of MO at different concentrations; (b) Standard absorption curve of MO; Table S1: Comparison of MO Removal Efficiency Between NiFe-LDH and Other Materials; Table S2: Removal amount and rate of NiFe-LDH; Figure S2: UV-visible absorption spectra of MO at different pH; Table S3: Chemical bond length of MO.
