# Supplementary material for: Contribution of Active Surface of NiFe-Layered Double Hydroxide on the Removal of Methyl Orange
Source: Materials (Basel). 2025 Feb 19;18(4):911. doi: 10.3390/ma18040911 (PMC11857282; doi:10.3390/ma18040911)
Supplement: Supplementary file 1 [file materials-18-00911-s001.zip › materials-3459227-supplementary.pdf]

Supporting information

Figure S1

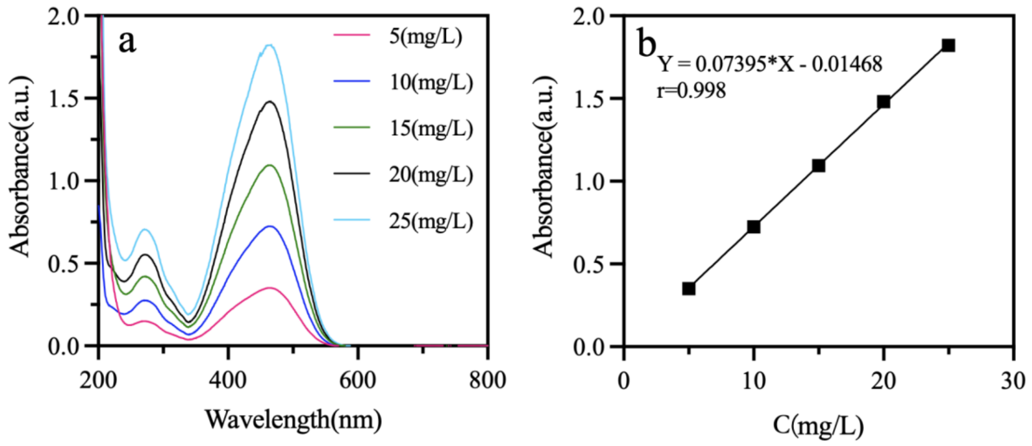

Figure S1 (a) Ultraviolet spectra of MO at different concentrations;(b) Standard absorption curve of MO

Table S1

Table S1 Comparison of MO Removal Efficiency Between NiFe-LDH and Other Materials

Material Type	Material	Time (min)	Removal Amount (mg/g)	Mechanism	Reference
Layered Double Hydroxides	NiFe-LDH	30	308.80	Adsorption	[20]
	MgAl-LDH	60	167.62	Adsorption	[21]
	ZnCr-LDH	120	104.08	Photocatalysis	[22]
Photocatalysts	TiO_2	120	60.20	Photocatalysis	[23]
	$g\text{-}C_3N_4$	40	9.10	Photocatalysis	[24]
Adsorbents	Activated Carbon	60	201.40	Physical adsorption	[25]
Advanced Oxidation	Fe^{2+}/H_2O_2 (Fenton)	180	212.50	·OH radical oxidation	[26]
	Co@GAC/PMS	25	96.40	SO_4^{-} • radical oxidation	[27]

Table S2

Table S2 Removal amount and rate of NiFe-LDH

C(mg/L)	Removal Rate(%)	$m_{wNiFeLDH-1}$(g)	Removal amount(mg/g)
50	97.04	0.01	118.63
150	77.45		284.05

250	60.67	370.84
350	50.87	435.32
450	40.80	503.91
550	37.65	506.63

Figure S2

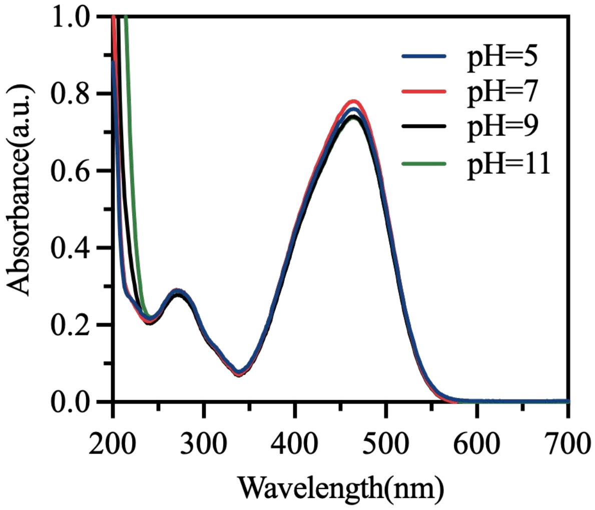

Figure S2 UV-visible absorption spectra of MO at different pH

Table S3

Table S3 Chemical bond length of MO

Bond	Length	Bond	Length	Bond	Length	Bond	Length
C3-S9	1.812	C19-C20	1.540	N25-C29	1.509	C20-H26	1.142
S9-O13	1.781	C20-C21	1.540	C6-N12	1.508	C28-H32	1.142
S9-O14	1.781	C21-C16	1.540	C16-N22	1.507	C28-H31	1.142
S9-O15	1.781	C5-C6	1.539	N12-N22	1.479	C4-H10	1.141
C2-C3	1.541	C6-C1	1.539	C29-H34	1.420	C1-H7	1.140
C1-C2	1.540	C17-C18	1.539	C29-H33	1.400	C2-H7	1.140
C3-C4	1.540	C16-C17	1.538	C29-H35	1.380	C5-H11	1.140
C4-C5	1.540	C19-N25	1.509	C28-H30	1.143	C18-H24	1.139
C18-C19	1.540	N25-C28	1.509	C17-H23	1.142	C21-H27	1.139

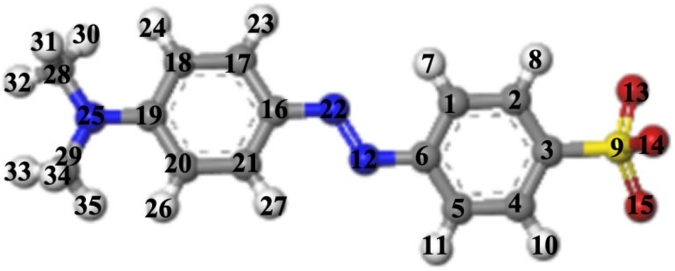
31 30
24
23
28
18 17
7
8
32
25 19
16 22
1 2
13
29
12 6
3 9 14
33 34
20 21
35
26
27
5 4
15
11
10
